# Establishing standardized evaluation protocols for peptide-target interaction prediction: the PTI-TAPE consensus framework eDelphi

**DOI:** 10.3389/fbinf.2026.1894725

**Published:** 2026-10-09

**Authors:** William J. Waldock, Guangshun Wang, Ara Darzi, Cesar de la Fuente-Nunez, Hutan Ashrafian, the PTI-TAPE Consortium Committee

**Affiliations:** 1 Imperial College London, Institute of Global Health Innovation, London, United Kingdom; 2 Department of Pathology, Microbiology, and Immunology, College of Medicine, University of Nebraska Medical Center, Omaha, NE, United States; 3 Machine Biology Group, Departments of Psychiatry and Microbiology, Perelman School of Medicine, Institute for Biomedical Informatics, Institute for Translational Medicine and Therapeutics, University of Pennsylvania, Philadelphia, PA, United States; 4 Departments of Bioengineering and Chemical and Biomolecular Engineering, School of Engineering and Applied Science, University of Pennsylvania, Philadelphia, PA, United States; 5 Department of Chemistry, School of Arts and Sciences, University of Pennsylvania, Philadelphia, PA, United States; 6 Penn Institute for Computational Science, University of Pennsylvania, Philadelphia, PA, United States

**Keywords:** benchmarking, eDelphi consensus, evaluation standards, machine learning, peptide-target interaction, PTI-TAPE

## Abstract

Computational peptide-target interaction (PTI) prediction has advanced rapidly as a tool for drug discovery, yet the field lacks agreed evaluation standards. Discrepancies in metrics, negative sampling strategies, and dataset construction across publications currently render cross-study comparisons methodologically intractable, ultimately impeding the clinical translation of therapeutic peptides. To resolve this, we present PTI-TAPE (Peptide-Target Interaction—Tasks Assessing Peptide Engagement), a standardised benchmarking framework developed via a structured three-round eDelphi consensus study with 15 international experts spanning computational biology, machine learning, and regulatory science. Operating under a robust consensus threshold (>80% agreement), the panel established 26 definitive standards across eight domains, governed by the new STRIDE (Standardisation, Transparency, Representativeness, Integration, Discovery, Evidence) framework. Key outcomes include mandating specific primary metrics for affinity and structure, enforcing a tiered negative sampling hierarchy that prioritizes experimental non-binders, and requiring mandatory temporal dataset splits. By providing a unified reporting checklist, benchmark specifications, and a biennial governance structure, PTI-TAPE ensures reproducible evaluation and fair method comparison, bridging the gap between computational PTI predictions and genuine therapeutic benefit.

## 1 Introduction

Peptides occupy a structurally privileged therapeutic niche, combining the binding specificity of large biologics with the synthetic accessibility of small molecules. More than eighty peptide therapeutics have received global approval, with over 170 candidates in clinical development (Fosgerau and Hoffmann, 2015; Muttenthaler et al., 2021). Deep learning architectures have demonstrated the capacity to accelerate every stage of the peptide drug pipeline, from interaction prediction through *de novo* design (Wan et al., 2024; Wong et al., 2023). AlphaFold 3 has enabled modelling of peptide-protein complexes with previously unattainable structural accuracy (Abramson et al., 2024), while protein language models trained at evolutionary scale have provided rich numerical representations that improved prediction across multiple downstream tasks (Elnaggar et al., 2022; Lin et al., 2023; Rives et al., 2021). Specialised architectures using graph neural networks (Kiouri et al., 2025; Kipf and Welling, 2017), SE(3)-equivariant frameworks (Fuc et al., 2020), and transformers adapted for molecular interaction tasks (Vaswani et al., 2017) have further expanded the methodological repertoire.

Despite this technical vitality, peptide-target interaction (PTI) prediction has been hampered by the absence of community-endorsed evaluation standards. The problem is systemic rather than incidental. Metrics as different as the area under the receiver operating characteristic curve (AUROC) (Hanley and McNeil, 1982), Matthews correlation coefficient (MCC) (Matthews, 1975), mean squared error, Spearman rank correlation (R^2^), and root mean square deviation (RMSD) have been employed as primary performance indicators across different publications without consensus on which metrics should be considered mandatory (Yang et al., 2020). Negative sampling strategies have varied across random, clustered, and sequence-similarity-filtered approaches, with more permissive methods producing substantially inflated performance estimates (Szymanski et al., 2012). This dependence on negative data is not peripheral; it determines whether reported performance reflects genuine molecular recognition. Since most repositories catalogue binders far more completely than confirmed non-binders, a model can attain high apparent accuracy by exploiting compositional or distributional artefacts of the negative set rather than learning the determinants of binding. The small-molecule virtual screening literature has shown repeatedly that property-matched or topology-preserving decoys are required to expose this failure mode, and that models trained on protein–ligand structures need not outperform ligand-only baselines once such controls are imposed. Comparable safeguards have been largely absent from peptide–target benchmarking, motivating the explicit negative-sampling provisions developed here. Dataset splits are infrequently documented; leakage from future data into training sets is therefore difficult to audit. Uncertainty quantification is inconsistently reported or absent (Lakshminarayanan et al., 2017). The consequence is that comparing two PTI prediction methods across independent benchmarks remains methodologically intractable, obscuring genuine advances and potentially distorting funding priorities (Paul et al., 2010; Wouters et al., 2020).

Community-driven consensus initiatives have resolved analogous evaluative crises in adjacent fields. The Critical Assessment of protein Structure Prediction (CASP) established blind prospective benchmarks that identified the most promising methodological directions across successive rounds (Pereira et al., 2021; Moult et al., 2018). The Critical Assessment of Functional Annotation (CAFA) demonstrated that agreement on evaluation methodology could advance a field characterised by annotation heterogeneity comparable to that in PTI prediction (Radivojac et al., 2013; Zhou et al., 2019). The Comparative Assessment of Scoring Functions (CASF) unified benchmarking in molecular docking and binding affinity estimation (Su et al., 2019; Li et al., 2014). Table 1 summarises the scope of these and other relevant initiatives relative to PTI prediction.

**TABLE 1:** Scope of relevant community benchmarking and reporting initiatives.

Initiative	Domain	Primary scope	Consensus mechanism	PTI coverage
CASP	Structural biology	Protein structure prediction	Blind prospective assessment	Partial (globular proteins only)
CAFA	Functional genomics	Protein function prediction	Community annotation challenge	No
CASF	Cheminformatics	Binding affinity scoring functions	Comparative scoring power evaluation	Partial (small molecules only)
CONSORT-AI	Clinical research	AI clinical trial reporting	Delphi/EQUATOR	No
TRIPOD + AI	Clinical prediction	ML prediction model reporting	Delphi/EQUATOR	No
STARD-AI	Diagnostics	AI diagnostic accuracy reporting	Delphi/EQUATOR	No
PTI-TAPE	Computational drug discovery	Peptide-target interaction benchmarking	eDelphi (this study)	Yes — primary scope

PTI prediction possesses characteristics that require domain-specific solutions. Peptide flexibility, particularly longer or linear sequences, complicates both structural prediction and docking in ways that have no direct counterpart in small-molecule benchmarking (Zorzi et al., 2017). The chemical diversity of therapeutic peptides, including linear, cyclic, lipidated, stapled, and non-natural amino acid-containing peptides, demands dataset coverage that existing benchmarking resources often do not provide (Lau and Dunn, 2018). High-quality experimental PTI data remain limited relative to the parameter dimensionality of leading models and are distributed across repositories with inconsistent curation standards (Agrawal et al., 2021; Wen et al., 2019; Liu et al., 2017). Biological interpretability also requires uncertainty quantification more rigorous than that routinely applied in small-molecule domains (Hirschf et al., 2020).

PTI-TAPE (Peptide-Target Interaction—Tasks Assessing Peptide Engagement) was conceived to address this gap. The initiative combined a systematic review and meta-analysis of existing PTI prediction benchmarking practices with a structured three-round eDelphi consensus study involving international experts identified for their contributions across computational peptide science, machine learning, drug discovery, and regulatory science.

## 2 Materials and methods

### 2.1 Study design and ethical governance

PTI-TAPE was designed as a multi-round expert consensus study employing eDelphi methodology, conducted under ethics approval from the Imperial College Research Governance and Integrity Team (reference 8059252). The eDelphi approach was selected because it provided a structured mechanism for aggregating expert opinion iteratively, reduced social influence biases that characterise in-person consensus meetings, and produced quantifiable measures of consensus trackable across rounds (Hasson and Keeney, 2011; Linstone and Turoff, 1975; Diamond et al., 2014). The method had established precedent in computational biology and clinical guideline development (Sounderajah et al., 2020; Schulz et al., 2010). The study was conducted over 10 weeks across three sequential rounds. All survey responses were collected through secure online platforms compliant with the UK Data Protection Act 2018 and the General Data Protection Regulation (Regulation EU, 2016). The study adhered to the Declaration of Helsinki (World Medical Association, 2013).

### 2.2 Participant recruitment and panel composition

A target panel of 25–30 international experts was established, consistent with guidance on panel size in Delphi methodology (Hasson and Keeney, 2011; Sinha et al., 2011). A total of 113 candidates were invited; 15 accepted and provided signed informed consent. Primary eligibility criteria required a minimum of five peer-reviewed publications in peptide-protein interactions, computational biology, machine learning for structural biology, or drug discovery, combined with publications within the preceding 5 years. Candidates were identified through systematic literature review of PTI prediction publications from 2019 to 2025, professional networks, and snowball sampling (Murphy et al., 1998). Secondary criteria targeted geographic representation (North America, Europe, Asia-Pacific), sectoral representation (academia, industry, regulatory bodies), career stage diversity, and methodological diversity. Exclusion criteria included absence of relevant domain expertise, research inactivity within the preceding 5 years, inability to commit to all three rounds, and conflicts of interest arising from significant commercial interests in specific PTI prediction methods.

### 2.3 Survey instrument design

Round 1 (3–4 weeks, estimated 45–60 min) deployed a comprehensive landscape mapping survey comprising Likert-scale ratings on a validated seven-point scale (Likert, 1932), item rankings, and open-ended qualitative questions. Domains covered included method maturity, evaluation metrics, dataset quality standards, negative sampling strategies, confidence reporting, barriers to standardisation, and research priorities.

Round 2 (2–3 weeks, estimated 30–45 min) presented anonymised, aggregated summaries of Round 1 responses, including median ratings, interquartile ranges, and thematic summaries. Participants were invited to reconsider their positions in light of collective input. Focus was directed toward items approaching but not yet reaching consensus and toward substantive disagreements. Dataset size items were reframed by task type (binary classification, affinity estimation, structural modelling) to address the task-dependence identified in Round 1.

Round 3 (2 weeks, estimated 20–30 min) presented near-final consensus standards for formal endorsement, solicited contributions to a prioritised implementation roadmap, and addressed governance structures.

### 2.4 Consensus definition and statistical analysis

An item was designated a consensus standard if it achieved agreement (ratings of six or seven) from ≥80% of responding participants, combined with low inter-respondent variance (Hasson and Keeney, 2011; Fitch et al., 2001). Items were classified as: Strong Consensus (median ≥6.0, IQR ≤1.0); Consensus (median ≥5.5, IQR ≤1.5); Near-Consensus (median ≥5.0 or IQR borderline); or No Consensus (median <5.0 or IQR >1.5). The RAND/UCLA Appropriateness Method (Fitch et al., 2001) informed threshold selection. Quantitative analysis employed descriptive statistics including median, mean, and interquartile range. Consensus evolution was tracked across rounds by comparing agreement proportions at each time point. Stability analysis assessed the consistency of individual ratings between rounds, with systematic shifts interpreted in the context of deliberative content. Subgroup analyses examined whether consensus levels differed across sectoral, geographic, or career-stage groupings. Qualitative data from open-ended responses underwent thematic analysis using a framework approach (Ritchie et al., 1994).

### 2.5 Data governance

Personal data were held separately from pseudonymised response data in secure environments and accessed only by the core study team. Survey responses were anonymised before aggregate analysis; individual data could not be retrospectively removed following withdrawal, a provision communicated in the participant information sheet. Data will be retained for a minimum of 10 years. International data transfers were governed by data processing agreements consistent with applicable data protection legislation (Regulation EU, 2016).

## 3 Results

### 3.1 Panel composition and response rates

A total of 113 experts were invited; 15 accepted and provided informed consent (acceptance rate 13.3%). Round 1 received 15 responses, Round 2 received 13 responses (87% retention), and Round 3 received 15 responses (100% of consented panel). The panel included expertise in computational biology, structural biology, machine learning, drug discovery, peptide chemistry, clinical research, and regulatory science, with representation from North America, Europe, and Asia-Pacific. The panel size falls at the lower bound of the target range (25–30) and below sample sizes in some comparable Delphi initiatives. The implications of this sample size for the strength of consensus claims are addressed in the Limitations section.

### 3.2 Round 1: landscape mapping

Experimental validation and binding affinity availability achieved strong consensus as essential data quality criteria (means 6.56 and 6.33 out of 7, respectively). The assertion that a centralised platform would improve reproducibility and facilitate fair comparisons also reached strong consensus (means 6.75 and 6.50). Limited training data were ranked the dominant technical challenge (mean rank 1.91 out of 9), while computational resource constraints were explicitly deprioritised (mean rank 7.64), indicating that data quality rather than compute capacity constitutes the primary bottleneck.

Binding affinity correlation emerged as the preferred quantitative standard (mean 6.25). No consensus was achieved for any binary classification metric: AUROC (mean 5.13, SD 1.73), AUPRC (mean 4.88, SD 1.81), F1 (mean 4.75, SD 1.67), and MCC (mean 4.63, SD 1.85) all received moderate but highly variable ratings with ranges spanning the full seven-point scale. This variability reflected genuine substantive disagreement rather than indifference, as discussed below.

A clear negative sampling hierarchy was established, with experimental non-binders ranked first (mean rank 1.27). However, a structural tension emerged: experimental non-binders were simultaneously the preferred strategy and the most severely underrepresented data type in existing repositories. Comprehensive documentation (mean 6.56) and open-source code (mean 6.38) were the highest-rated accessibility features.

Responses to dataset size items spanned five orders of magnitude (from fewer than 100 to more than 1,000,000 binding pairs), reflecting genuine task-dependence: respondents with structural modelling expertise proposed substantially smaller thresholds than those working primarily in classification, an observation that informed the task-stratified reframing applied in Round 2.

### 3.3 Round 2: convergence and reframing

Round 2 re-presented unresolved items as structured statements with anonymised Round 1 distributions. R^2^ or Spearman correlation achieved the highest rating across all items as the required primary metric for affinity regression (mean 6.55, up from 6.25 in Round 1), and RMSD was confirmed as required for structure-based predictions (mean 6.18, up from 5.75). Both reached consensus.

Binary classification metrics remained below threshold but showed narrowing disagreement relative to Round 1. AUROC rose from mean 5.13 to 5.45 (SD reduced from 1.73 to 1.37); AUPRC rose from 4.88 to 5.55 (SD reduced from 1.81 to 1.21); MCC rose from 4.63 to 5.45 (SD reduced from 1.85 to 1.29). The convergence pattern, with rising means and declining variance, indicated that Round 1 feedback shifted opinion toward agreement rather than merely hardening positions, consistent with the effective deliberative function of the eDelphi process.

Target diversity across multiple protein families (mean 6.27) and a minimum structural resolution of 2.5 Å (mean 6.18) achieved consensus. A minimum of 10,000 binding pairs for training sets received moderate agreement (mean 5.45) but remained near-consensus. The task-stratified reframing introduced in this round, which distinguished thresholds for classification, affinity estimation, and structural modelling, substantially reduced the variance that had characterised Round 1 responses.

Standardised benchmark datasets were unanimously ranked the highest-priority platform feature (mean rank 1.00, SD 0.00). Pre-trained baseline models achieved strong consensus (mean 6.27), and a 2-year review cycle with versioned releases was strongly endorsed (mean 6.30). Governance by an elected, rotating multi-disciplinary steering committee reached consensus (mean 5.91). Journal and funding agency compliance mandates were rated favourably but with wider dispersion (mean 5.82, SD 1.34). Cloud computing did not reach consensus (mean 4.45), consistent with low prioritisation across both rounds.

### 3.4 Round 3: final ratification

Round 3 focused on final adjudication of remaining items and STRIDE ratification. Strong consensus was achieved for endorsing STRIDE as the governing framework for PTI Evaluation Standard v1.0. Panellists affirmed STRIDE as balanced and fit for purpose, though several noted limitations including the lack of realistic negatives in existing datasets, incomplete coverage of temporal splits, affinity prediction, uncertainty estimation, and peptide-specific diversity handling. These comments reinforced the need for PTI-TAPE rather than undermining the framework, as PTI-TAPE was designed specifically to address these gaps.

AUPRC as a co-primary metric alongside AUROC for imbalanced tasks achieved consensus (mean 6.09, up from 5.55 in Round 2), resolving the persistent disagreement from prior rounds. The resolution was driven by two factors visible in the deliberative record: first, Round 2 feedback presented evidence that AUROC alone provides an incomplete picture of discriminative performance under realistic class imbalance conditions (Davis and Goadrich, 2006), and second, the reframing of AUPRC as a co-primary rather than alternative metric reduced the perceived trade-off that had driven earlier disagreement. MCC was endorsed as recommended but not mandatory.

Minimum dataset sizes were reframed as task-stratified recommendations rather than universal requirements, reconciling the longstanding tension between machine learning and experimental perspectives. Journal and funding agency compliance narrowed to consensus, with the majority supporting a phased approach progressing from encouragement to requirement. The complete set of 26 ratified standards across 8 domains is presented in Table 2. Each principle of STRIDE is defined below and mapped to specific checklist items in Table 3. STRIDE was not adopted from an existing reporting standard; it was formulated within this study as the organising structure for the ratified Standards and endorsed by the panel in Round 3. Its positioning relative to prior community initiatives (none of which provide primary coverage of peptide–target interaction benchmarking) is set out in Table 1, which contrasts each initiative by domain, primary scope, consensus mechanism, and PTI coverage.

**TABLE 2:** PTI-TAPE Framework Standards 26 standards across 8 domains.

#	Domain	Standard	Applicability	Round 1	Round 2	Round 3
1	Data quality	Report the experimental validation method used to confirm interaction status for all positive training, validation, and test pairs	All tasks	Strong consensus (6.56)	—	—
2	Data quality	State the source databases for all interaction data, including version numbers and access dates	All tasks	Strong consensus	—	—
3	Data quality	Report binding affinity values (Kd, Ki, or IC50) for all quantitative affinity estimation datasets	Affinity estimation	Strong consensus (6.33)	—	—
4	Data quality	Report target protein family coverage and stratification across peptide length, secondary structure class, cyclisation state, and chemical modification	All tasks	Consensus (5.88)	Consensus (6.27)	—
5	Data quality	State the crystallographic or cryo-EM resolution threshold; recommended minimum is 2.5 Å	Structure modelling	Near-consensus (5.13)	Consensus (6.18)	—
6	Data quality	Report sequence identity clustering thresholds using 30%, 50%, and 90% cutoffs	All tasks	Near-consensus (5.00)	Consensus (5.55)	—
7	Dataset construction	Apply temporal splits to all benchmark datasets	All tasks	Consensus	—	—
8	Dataset construction	Report absolute dataset sizes, stratified by task type. Minimum 10,000 binding pairs recommended for binary classification	All tasks	No consensus	Near-consensus (5.45)	Consensus (task-stratified)
9	Negative sampling	Apply experimental non-binders as the primary negative sampling strategy with defined fallback hierarchy	Classification	Strong consensus (1.27)	—	—
10	Negative sampling	Report the negative sampling strategy employed with biological rationale for any departure	Classification	Strong consensus	—	—
11	Evaluation metrics	Report binding affinity correlation using both Pearson and Spearman coefficients, with RMSE, each accompanied by 95% confidence intervals. Studies must additionally demonstrate correct rank ordering across the full affinity range, from high-affinity binders through to confirmed non-binders	Affinity estimation	Consensus (6.25)	Consensus (6.55)	—
12	Evaluation metrics	Report structural accuracy as ligand RMSD from crystallographic reference	Structure modelling	Consensus (5.75)	Consensus (6.18)	—
13	Evaluation metrics	Report AUROC as a required primary metric for all binary PTI classification benchmarks	Classification	Near-consensus (5.13)	Near-consensus (5.45)	Consensus
14	Evaluation metrics	Report AUPRC as a co-primary metric alongside AUROC for imbalanced tasks	Classification	Near-consensus (4.88)	Near-consensus (5.55)	Consensus
15	Evaluation metrics	Report MCC as a recommended supplementary metric	Classification	No consensus	Near-consensus (5.45)	Recommended
16	Confidence reporting	Include calibrated confidence scores and uncertainty estimates with all predictions	All tasks	Consensus	Reinforced	—
17	Transparency	Provide comprehensive documentation enabling independent reproduction	All tasks	Strong consensus (6.56)	—	—
18	Transparency	Release all benchmark evaluation code as open-source software	All tasks	Strong consensus (6.38)	—	—
19	Transparency	Provide tutorial and training materials for researchers new to PTI benchmarking	All tasks	Consensus (6.11)	—	—
20	Transparency	Provide API access to evaluation infrastructure	All tasks	Consensus (5.88)	—	—
21	Transparency	Release pre-trained baseline models for each task category	All tasks	Consensus (5.50)	Strong consensus (6.27)	—
22	Statistical Testing	Report statistical significance of performance comparisons using Wilcoxon signed-rank or bootstrap resampling	All tasks	Consensus	—	—
23	Open science	Make all benchmark datasets publicly available under CC BY 4.0, compliant with FAIR data principles	All tasks	Consensus	—	—
24	Open science	Disclose any commercial interests or conflicts of interest	All tasks	Consensus	—	—
25	Governance	Follow a formal 2-year review cycle with versioned releases	All tasks	New — round 2	Strong consensus (6.30)	—
26	Governance	Journals and funding agencies should require compliance, with phased implementation	All tasks	New — round 2	Near-consensus (5.82)	Consensus

“—” indicates no change from previous round (item already resolved). STRIDE principles ratified as governing framework in Round 3.

**TABLE 3:** The STRIDE principles governing PTI-TAPE.

Principle	Definition	Primary checklist items
Standardisation	Uniform evaluation protocols applied identically across all submitted methods	7, 8, 9, 10, 14, 15, 23
Transparency	Full disclosure of methods, data, code, and limitations enabling independent reproduction	18, 19, 20, 21, 22
Representativeness	Balanced dataset coverage across peptide biology, chemistry, structure, and target diversity	4, 5, 6, 7
Integration	Connecting computational predictions to experimental validation frameworks and clinical contexts	1, 2, 3, 17
Discovery	Enabling methodological innovation while maintaining rigorous comparative assessment	16, 22, 24
Evidence	Alignment with clinical utility and regulatory requirements for computational drug discovery	12, 13, 25, 26

Standardisation requires that evaluation protocols be applied identically across all submitted methods, eliminating variability arising from *ad hoc* methodological choices. Transparency mandates full disclosure of methods, data, code, and limitations sufficient to enable independent reproduction. Representativeness requires balanced dataset coverage across peptide biology, chemistry, structure, and target diversity, ensuring that benchmarks do not inadvertently favour methods optimised for narrow subsets of the interaction space. Integration connects computational predictions to experimental validation frameworks and clinical contexts, reflecting the principle that benchmark performance must ultimately be interpretable in terms of translational utility. Discovery ensures that the framework enables methodological innovation rather than constraining it, maintaining rigorous comparative assessment without prescribing model architectures. Evidence aligns evaluation with clinical utility and regulatory requirements for computational drug discovery, recognising that the long-term value of PTI prediction depends on its acceptability within regulatory and clinical decision-making processes.

The STRIDE principles were presented to the expert panel in Round 3, where they achieved strong consensus as the governing framework for PTI Evaluation Standard v1.0. Panellists affirmed STRIDE as balanced and fit for purpose. The most frequently requested enhancement was the provision of worked examples demonstrating compliance with each component, which will be developed as part of the platform documentation (Table 4).

**TABLE 4:** Rationale for Key new or modified standards.

Item	Rationale
10 (negative sampling hierarchy)	Biologically informed negative sampling was employed in fewer than one-quarter of prior studies; random pairing demonstrably inflates AUROC estimates. A tiered hierarchy with experimental non-binders as gold standard provides a practically implementable standard acknowledging data availability constraints
7 (temporal splits)	Data leakage from future interaction data into training sets was difficult to audit due to absent reporting standards. Temporal splits represent prospective evaluation conditions and prevent chronological contamination
17 (confidence calibration)	Uncertainty quantification was inconsistently reported or absent. Calibrated confidence scores are essential for experimental scientists making resource-allocation decisions based on computational predictions
8–9 (dataset size, stratified)	Round 1 responses spanned five orders of magnitude, reflecting genuine task-dependence. Stratification by task type produced actionable minimum thresholds for each evaluation context
25 (Living benchmark versioning)	The rapidly evolving nature of PTI prediction methods necessitates annual benchmark review cycles. Versioning enables transparent tracking of how standards evolve over time

## 4 Discussion

The systematic review underlying PTI-TAPE documented a field that had developed not merely heterogeneous evaluation standards but, in many respects, no coherent standards at all. The diversity of metrics, inconsistency of dataset practices, and near-complete absence of uncertainty quantification collectively constituted scientific infrastructure poorly equipped to support clinical translation (Schneider et al., 2020; Stokes et al., 2020). The consensus findings of this eDelphi reveal a field at an important methodological juncture. The rapid convergence on binding affinity correlation (Spearman/R^2^) and structural RMSD as primary metrics reflects alignment around outputs most proximal to experimental utility. By contrast, the protracted disagreement over classification metrics, which required three rounds to resolve, reflects genuine substantive tension. The eventual consensus on AUROC plus AUPRC as co-primary metrics, with MCC recommended, represents a principled resolution: AUROC captures overall discriminative power while AUPRC addresses the sensitivity to class imbalance that renders AUROC alone insufficient under realistic conditions (Hanley and McNeil, 1982; Davis and Goadrich, 2006). The convergence pattern across rounds, with rising means and declining variance, demonstrates that iterative deliberation with anonymised feedback functioned as intended, shifting opinions through evidence rather than social pressure.

The negative sampling hierarchy constitutes one of the most consequential outputs of the consensus process. The expert panel’s strong preference for experimental non-binders as the gold standard, combined with their simultaneous recognition that such data are critically underrepresented, produced a practical resolution: a tiered fallback hierarchy comprising experimental non-binders, literature-derived non-binding peptides, structural non-compatible pairs, and shuffled sequences. This hierarchy acknowledges data availability constraints while establishing a clear quality gradient. It provides an implementable standard that prior benchmarking frameworks had not addressed for the peptide domain.

Beyond ranking these sources, three practical requirements follow. First, the provenance of negatives must be reported as explicitly as that of positives: studies should state how non-binders were obtained or generated, since the lowest tier (sequence shuffling, which preserves amino-acid composition while disrupting the determinants of engagement) is conceptually analogous to the property-matched decoys used in small-molecule screening and carries the same risk of introducing separable artefacts if applied naively. Second, the quantity of negatives and their ratio to positives must be declared, because metric values are not interpretable without them; a benchmark dominated by trivially distinguishable negatives will report inflated discrimination irrespective of model quality. Third, negatives should be used not merely to populate a classification task but to test monotonic scoring across the affinity continuum, such that high-affinity binders are scored above moderate binders, moderate above weak, and weak above confirmed non-binders. Only consistent ordering of this kind, across diverse targets and a wide affinity range, provides evidence that a method has captured the physics of association rather than dataset-specific structure.

The dataset size debate illustrates a productive tension between subdisciplinary perspectives. Round 1 responses spanning five orders of magnitude were not noise; they reflected genuine differences in data requirements across binary classification (where large datasets are standard), affinity estimation (where high-quality binding constants are scarce), and structural modelling (where even hundreds of well-resolved complexes may suffice). The task-stratified reframing introduced in Round 2 substantially reduced variance and ultimately achieved consensus, demonstrating that apparent disagreement can resolve when the question is posed at the appropriate level of specificity.

### 4.1 Limitations

Several limitations require forthright acknowledgement. First, the panel of 15 consented experts, while internally diverse, falls at the lower bound of the target range and below sample sizes in some comparable initiatives. With 13 respondents in Round 2, a single participant shifting from 5 to 7 on the Likert scale moves the mean by approximately 0.15, which is material relative to the gap between consensus categories. The consensus claims made here should be interpreted as reflecting the views of a carefully selected but small expert group rather than the field at large, and the standards would benefit from broader community validation through open comment periods and prospective adoption studies. We note, however, that the validity of a Delphi process derives from the stability and convergence of expert judgement rather than from sample representativeness. The rising means and falling variance observed across rounds for the contested classification metrics, together with full panel retention at Round 3, indicate genuine deliberative convergence rather than entrenchment of initial positions.

Second, the panel skewed towards computational and academic fields relative to the intended composition. Experimental peptide chemists, clinical investigators, and regulatory scientists were underrepresented despite targeted recruitment. Domain-specific considerations for antimicrobial peptide design (Wang et al., 2016; Torres et al., 2018), cyclic peptide optimisation (Zorzi et al., 2017), and peptide hormone pharmacology may consequently be underweighted. Snowball sampling and professional society outreach were employed to mitigate this imbalance, but the composition inevitably shapes which standards emerge and which do not.

Third, eDelphi methodology, despite its structural safeguards against social influence, cannot eliminate shared intellectual bias in a community whose leading figures often share methodological training and institutional networks (Linstone and Turoff, 1975; Murphy et al., 1998). The standards may therefore reflect the assumptions of a particular intellectual tradition within computational biology.

Fourth, this study reports consensus achieved through deliberation but cannot demonstrate that adoption of the resulting standards improves real-world outcomes. Prospective evaluation, including comparisons of reproducibility and translational success between studies that adhere to PTI-TAPE standards and those that do not, represents a necessary next step. Accordingly, the present work should be read as establishing the consensus standard; demonstrating that adherence improves reproducibility and ranking stability is a separate, prospective undertaking.

### 4.2 PTI-TAPE: analysis, applications, and future directions

#### 4.2.1 Living platform and future directions

PTI-TAPE was designed as a living platform rather than a static document. Biennial standard reviews, supported by interim benchmark-data updates where necessary, will be coordinated by the community steering committee established through Round 3.

These updates will incorporate new experimental interaction data, accommodate novel prediction modalities including generative design paradigms, and respond to the evolving regulatory landscape for computational methods (US Food and Drug Administration, 2021; Topol, 2019). Integration of experimental validation datasets from high-throughput binding assays and cryo-EM structural analysis offers a route to expanding benchmark coverage while maintaining quality standards (Senior et al., 2020; Berman et al., 2000). The potential for PTI-TAPE standards to support regulatory submissions represents the most consequential long-term application, requiring close coordination with regulatory science expertise (US Food and Drug Administration, 2021).

PTI-TAPE does not aim to constrain methodological innovation or prescribe model architectures. Its scope is limited to evaluation standards: what is assessed, how it is measured, and under what conditions. The framework does not address standards for clinical decision support, regulatory approval pathways for AI/ML-based software as a medical device, or the design of prospective experimental validation studies, each of which requires separate community consensus. The framework further guards against a common reporting artefact: correlations computed over few points spanning a narrow affinity range, which can appear strong yet carry no evidence of true ranking ability. Standard 11 mandates demonstrated rank ordering across the full spectrum, rendering such reports non-compliant.

### 4.3 Critical analysis of framework architecture

#### 4.3.1 Consensus dynamics and stability

The progression of Standards 8, 13, 14, and 26 from contested to ratified status across three rounds reveals that consensus timelines correlate with the degree to which proposed standards implicitly critique established practice (Diamond et al., 2014; Humphrey-Murto et al., 2017a). Standard eight achieved consensus only after accepting task-stratified thresholds, illustrating how benchmark design accommodates legitimate heterogeneity across prediction tasks (Humphrey-Murto et al., 2017b). Standards 13 and 14, mandating AUROC and AUPRC as co-primary classification metrics, encountered sustained resistance from groups whose methods had been validated exclusively against accuracy-based measures (Saito and Rehmsmeier, 2015; Davis and Goadrich, 2006). Standard 26, requiring journal and funder compliance, proved most contentious, with panellists raising concerns about enforcement feasibility and the risk of premature mandates stifling emerging research groups (Hasso et al., 2000).

Standards most vulnerable to future challenge include Standard 15, which achieved only recommended status for Matthews Correlation Coefficient (Chicco and Jurman, 2020), and Standard 6, whose sequence identity clustering thresholds may face pressure as protein language model embeddings render sequence identity an incomplete proxy for functional similarity (Elnaggar et al., 2022; Lin et al., 2023). The steering committee should institute formal tracking of emerging dissent, modelled on living systematic review paradigms in clinical evidence synthesis (Elliott et al., 2017).

#### 4.3.2 Metric coherence

The co-requirement of AUROC and AUPRC for classification (Standards 13–14) addresses the well-documented insensitivity of AUROC to class imbalance (Saito and Rehmsmeier, 2015; Chicco et al., 2021), but the framework does not specify a minimum imbalance ratio below which AUPRC becomes the *de facto* primary metric, leaving room for selective optimisation (Boughorbel et al., 2017). For affinity estimation, Pearson and Spearman correlation alongside Root Mean Squared Error (RMSE) (Standard 11) is methodologically sound but insensitive to systematic bias; adding a concordance correlation coefficient requirement in a future version would address this gap (Lin, 1989). For structural modelling, ligand RMSD (Standard 12) does not assess whether predicted binding modes reproduce experimentally observed interaction fingerprints at the residue level; interaction pattern similarity metrics would strengthen translational relevance (Deng et al., 2004; Salentin et al., 2015).

#### 4.3.3 Governance sustainability

Three primary failure modes threaten the governance architecture. First, volunteer fatigue within the steering committee, a risk supported by open-source governance studies showing engagement decline after the second review cycle (O'Mahony and Ferraro, 2007). Second, the phased enforcement model (Standard 26) assumes journal and funder convergence without a coordinating mechanism; CONSORT and ARRIVE precedents demonstrate adoption timelines spanning a decade with inconsistent enforcement (Moher et al., 2010; Percie du Sert et al., 2020). Third, inter-version comparability (Standard 25) lacks a concrete technical specification for handling deprecated standards in longitudinal comparisons. Safeguards should include formal memoranda with major journals establishing shared compliance timelines (Simera et al., 2010), a machine-readable versioning schema enabling automated compliance checking, and tiered committee membership funded through benchmark submission fees (Weber et al., 2019).

#### 4.3.4 Scope boundaries and the last-mile problem

The exclusion of clinical decision support, regulatory pathways, and prospective validation from PTI-TAPE’s scope avoids the regulatory complexities that have delayed other AI/ML standardisation efforts (Muehlematter et al., 2021) but creates a translational discontinuity: exemplary benchmark performance does not demonstrate suitability for any specific clinical or regulatory application (Kelly et al., 2019). The FDA’s evolving AI/ML framework emphasises prospective validation and continuous monitoring as prerequisites for regulatory clearance (US Food and Drug Administration, 2021; US Food and Drug Administration, 2023), and the absence of an explicit bridge between benchmark evaluation and regulatory evidence requirements may lead to misinterpretation of PTI-TAPE compliance as regulatory readiness (Wu et al., 2021). The framework should include a prominent caveat specifying this relationship, developed in consultation with regulatory science organisations. To prevent over-interpretation, we state explicitly that PTI-TAPE is a community evaluation and reporting standard intended for method developers and the research community; it neither constitutes nor implies regulatory endorsement. Compliance documents the methodological rigour of a benchmark, not the regulatory readiness of a method. Given the limited regulatory participation in this panel, the standards should be regarded as informing, rather than pre-empting, future regulatory consultation.

#### 4.3.5 STRIDE principle tensions

The most consequential conflict exists between Standardisation and Discovery. Methods employing novel prediction paradigms, such as generative models producing binding peptide distributions, are poorly served by metrics designed for discriminative classifiers (Ingraham et al., 2019); a formal Innovation Track permitting adapted evaluation protocols would address this tension while maintaining standardised baselines. Representativeness and data availability conflict because the experimentally characterised interaction space is heavily biased toward therapeutically relevant targets and linear peptides made using 20 standard amino acids (Muttenthaler et al., 2021; Wang et al., 2016; Cunningham et al., 2017), and achieving representative coverage may require computationally generated data that introduces circularity concerns. Evidence and Integration jointly demand translational interpretability but provide no mechanism for weighting benchmark tasks by clinical relevance.

### 4.4 Most consequential applications

#### 4.4.1 Regulatory submission support

PTI-TAPE compliance could serve as a credibility signal within Investigational New Drug (IND) and Biologics License Application (BLA) submissions relying on computational candidate identification, as regulatory agencies have signalled willingness to consider computational evidence provided methods are transparently validated (US Food and Drug Administration, 2021; European Medicines Agency, 2024). A six-month consultation with regulatory science experts should map PTI-TAPE standards onto existing evidentiary frameworks. By eighteen months, pilot submissions from pharmaceutical sponsors should incorporate PTI-TAPE compliance documentation. By thirty-six months, formal agency engagement through pre-submission meetings should establish PTI-TAPE as a recognised reference standard (Vamathevan et al., 2019). The primary obstacle is regulatory conservatism; mitigation requires sustained engagement with regulatory science organisations and agency-sponsored workshops.

#### 4.4.2 High-throughput experimental integration

Convergence of phage display, mRNA display, and microarray-based screening with computational prediction creates an opportunity for PTI-TAPE to bridge computational and experimental workflows (Muttenthaler et al., 2021; Rogers et al., 2018). Partnerships with experimental groups should define PTI-TAPE-compatible data exchange formats within 6 months. A pilot integration study comparing benchmarked predictions against at least 10,000 experimentally validated pairs should be completed by 18 months. By 36 months, an automated pipeline linking benchmark submissions to experimental validation queues should be operational. Data quality heterogeneity across platforms is the primary risk; mitigation requires minimum assay quality standards aligned with Standard 1.

#### 4.4.3 Journal and funder adoption

Mandatory PTI-TAPE compliance would eliminate *ad hoc* benchmark selection and favourable cherry-picking of evaluation conditions (Weber et al., 2019; Boulesteix et al., 2013). Within 6 months, editorial board endorsement from five major journals should establish encouragement-level recommendations (Simera et al., 2010). By eighteen months, at least two journals should implement mandatory compliance checklists analogous to CONSORT and PRISMA requirements (Moher et al., 2010; Page et al., 2021). By thirty-six months, at least one funding agency should require PTI-TAPE documentation in grant progress reports. The coordination problem among journals, each facing competitive pressure not to impose requirements, requires simultaneous multi-journal engagement and learned society endorsement (Nosek et al., 2015).

#### 4.4.4 CRO procurement qualification

Pharmaceutical companies lack standardised criteria for evaluating vendor-offered PTI modelling capabilities (Vamathevan et al., 2019). A PTI-TAPE compliance certification programme should be designed within six months, with pilot adoption by pharmaceutical companies in vendor qualification processes by eighteen months. Industry consortium endorsement through organisations such as the Pistoia Alliance should formalise PTI-TAPE as a vendor evaluation standard by thirty-six months. Resistance from CROs whose proprietary methods perform poorly on standardised benchmarks is the primary risk; framing compliance as quality assurance rather than competitive ranking mitigates this concern.

#### 4.4.5 Living leaderboard with automated compliance scoring

A publicly accessible leaderboard would provide transparent, objective method comparison (Weber et al., 2019). A minimum viable platform supporting automated submission and compliance verification for Data Quality and Transparency standards should be delivered within six months. Full 26-standard coverage with automated non-compliance flagging should be operational by eighteen months. By thirty-six months, temporal tracking across benchmark versions should enable longitudinal progress analysis supporting the biennial review process. Gaming through benchmark overfitting is the primary risk; periodically refreshed held-out evaluation sets and mandatory containerized submission could mitigate this threat (Boulesteix et al., 2013).

## 5 Conclusion

PTI-TAPE addresses a foundational deficiency in the infrastructure of computational drug discovery. Through a systematic review, a structured three-round eDelphi consensus study with 15 international experts, and a principled platform architecture governed by the STRIDE framework, the initiative produced 26 evaluation standards across 8 domains that are technically rigorous, endorsed by the expert panel, and designed for practical implementation. The full reporting checklist (Table 5) and development process (Table 6) are provided to enable immediate adoption. The field of peptide-target interaction prediction has developed remarkable technical capabilities; PTI-TAPE provides the evaluative infrastructure required to translate those capabilities reliably into therapeutic benefit.

**TABLE 5:** PTI-TAPE Abstracts Checklist for use when reporting PTI prediction benchmarking studies in journal or conference abstracts.

Item	Checklist item
1	Identify the study as a PTI benchmarking evaluation and state the task type (classification, affinity estimation, structural modelling)
2	State the source database(s) and dataset sizes for the test partition
3	Describe the negative sampling strategy applied
4	State the temporal split cutoff date or data provenance period
5	Report the primary performance metric(s) with confidence intervals
6	State whether calibrated confidence scores were provided
7	Identify the baseline comparator method(s) used for significance testing
8	State whether code and datasets are publicly available
9	Report any conflicts of interest relevant to the evaluated method
10	State the principal conclusion regarding benchmarked method performance

**TABLE 4:** PTI-TAPE development process.

Stage	Phase	Action	Participants	Output
1	Systematic review	Systematic review and meta-analysis of PTI benchmarking practices across MEDLINE, Embase, and bioRxiv (2015–2025)	W.J.W., H.A.	Identification of metric heterogeneity, negative sampling inconsistency, and absence of uncertainty quantification standards
2	Candidate item generation	Project team extraction of evaluation domains and candidate standards	Project team (W.J.W., H.A., A.D., G.W., C.d.l.F.-N.)	55 candidate items across 7 evaluation domains
3	Expert recruitment	Literature review (2019–2025) + snowball sampling; eligibility: ≥5 publications, active within 5 years	Target: 25–30; invited: 113; accepted: 15	Confirmed expert panel with signed informed consent
4	Round 1	Landscape mapping; 3–4 weeks; 55 items rated	Respondents: 15	Strong consensus: 8 items; No consensus: Dataset size, classification metrics; 5 new items *via*qualitative analysis
5	Round 2	Consensus building; 2–3 weeks; anonymised round 1 results presented	Participants: 13; items carried forward: 21; new items: 5	Refined consensus; resolution of near-consensus items; governance model preference established
6	Round 3	Final ratification; 2 weeks; 26 items for ratification	Participants: 15	PTI evaluation standards v1.0 endorsed; implementation roadmap established; governance ratified
7	Dissemination	Manuscript submission; platform release; governance committee activation	Community steering committee	PTI-TAPE published; annual update cycle initiated

Consensus classification: Strong Consensus = median ≥6.0, IQR ≤1.0; Consensus = median ≥5.5, IQR ≤1.5; Near-Consensus = median ≥5.0 or IQR, borderline; No Consensus = median <5.0 or IQR >1.5. Ethics approval: Imperial College London, reference 8059252.

PTI-TAPE’s long-term impact depends on the institutional, regulatory, and community infrastructure supporting implementation. The consensus dynamics across three Delphi rounds demonstrate sufficient legitimacy to anchor a community-wide evaluation paradigm, but sustaining that legitimacy requires proactive governance and adaptive responsiveness (Diamond et al., 2014; Humphrey-Murto et al., 2017a). The five applications outlined above create a self-reinforcing ecosystem in which regulatory credibility, journal adoption, vendor qualification, experimental integration, and community transparency each amplify the others, establishing PTI-TAPE as the definitive evaluation infrastructure for peptide–target interaction prediction.

## Data Availability

The original contributions presented in the study are included in the article/supplementary material, further inquiries can be directed to the corresponding author.

## PTI-TAPE Consortium Committee

Anna Barnard, Rebecca Birolo, Mark Blaskovich, Carlos A. Brizuela, Miguel Augusto Rico Botas Castanho, Marco Calvinho Cavaco, Renee Fleeman, Dhira Joshi, Daniela Kalafatovic, Goran Mausa, José L. Medina-Franco, Arun Prasad Pandurangan, Fabien Plisson, Cláudio Manuel Soares and Jianyang Zeng.
